# Dietary Supplementation with Biobran/MGN-3 Increases Innate Resistance and Reduces the Incidence of Influenza-like Illnesses in Elderly Subjects: A Randomized, Double-Blind, Placebo-Controlled Pilot Clinical Trial

**DOI:** 10.3390/nu13114133

**Published:** 2021-11-19

**Authors:** Ahmed F. Elsaid, Sudhanshu Agrawal, Anshu Agrawal, Mamdooh Ghoneum

**Affiliations:** 1 Department of Community Medicine and Public Health, Zagazig University, Zagazig 44519, Egypt; 2 Division of Basic and Clinical Immunology, Department of Medicine, University of California, Irvine, CA 92697, USA; sagrawal@uci.edu (S.A.); aagrawal@uci.edu (A.A.); 3 Department of Surgery, Charles R. Drew University of Medicine and Science, Los Angeles, CA 90059, USA; mghoneum@ucla.edu

**Keywords:** Biobran/MGN-3, old adults, influenza-like illness, NK cell activity, RIG-1, MDA5, ISG-15, MX1, degranulation assay, flow cytometry

## Abstract

Influenza-like illness (ILI) remains a major cause of severe mortality and morbidity in the elderly. Aging is associated with a decreased ability to sense pathogens and mount effective innate and adaptive immune responses, thus mandating the development of protective nutraceuticals. Biobran/MGN-3, an arabinoxylan from rice bran, has potent anti-aging and immunomodulatory effects, suggesting that it may be effective against ILI. The objective of the current study was to investigate the effect of Biobran/MGN-3 on ILI incidence, natural killer (NK) cell activity, and the expressions of RIG-1 (retinoic acid-inducible gene 1), MDA5 (melanoma differentiation-associated protein 5), and their downstream signaling genes ISG-15 (interferon-stimulated genes 15) and MX1 (myxovirus (influenza) resistance 1, interferon-inducible). A double-blind, placebo-controlled clinical trial included eighty healthy older adults over 55 years old, 40 males and 40 females, who received either a placebo or Biobran/MGN-3 (500 mg/day) for 3 months during known ILI seasonality (peak incidence) in Egypt. The incidence of ILI was confirmed clinically according to the WHO case definition criteria. Hematological, hepatic, and renal parameters were assessed in all subjects, while the activity of NK and NKT (natural killer T) cells was assessed in six randomly chosen subjects in each group by the degranulation assay. The effect of Biobran/MGN-3 on RIG-1 and MDA5, as well as downstream ISG15 and MX1, was assessed in BEAS-2B pulmonary epithelial cells using flow cytometry. The incidence rate and incidence density of ILI in the Biobran/MGN-3 group were 5.0% and 0.57 cases per 1000 person-days, respectively, compared to 22.5% and 2.95 cases per 1000 person-days in the placebo group. Furthermore, Biobran/MGN-3 ingestion significantly enhanced NK activity compared to the basal levels and to the placebo group. In addition, Biobran/MGN-3 significantly upregulated the expression levels of RIG-1, MDA5, ISG15, and MX1 in the human pulmonary epithelial BEAS-2B cell lines. No side effects were observed. Taken together, Biobran/MGN-3 supplementation enhanced the innate immune response of elderly subjects by upregulating the NK activity associated with reduction of ILI incidence. It also upregulated the intracellular RIG-1, MDA5, ISG15, and MX1 expression in pulmonary epithelial tissue cultures. Biobran/MGN-3 could be a novel agent with prophylactic effects against a wide spectrum of respiratory viral infections that warrants further investigation.

## 1. Background

Influenza-like illness (ILI) is a viral infection with characteristic annual seasonality that leads to around 3–5 million severe illnesses and an estimated 500,000 annual deaths around the world [1,2]. The World Health Organization (WHO) has recently revised the definition of ILI to be “acute respiratory illness with a measured temperature of ≥38 °C and cough, with onset within the past 10 days” [3]. The diagnosis of influenza itself cannot be made without virological confirmation, which is costly and rarely performed in public health surveillance systems. To enhance the sensitivity of influenza surveillance systems to capture influenza, ILI has been recommended by the WHO to be used as a surrogate for influenza infection [4]. The simple clinical criteria used to define ILI was associated with low specificity. Therefore, in most surveillance systems around the world, a wide array of viruses has been isolated from ILI cases, including the respiratory syncytial virus, metapneumovirus, corona viruses, adenoviruses, rhinoviruses, and others [4,5,6,7]. In Egypt, during the period from 2012 to 2015, influenza viruses were isolated from only 13% of ILI cases identified in 13 different sentinel surveillance sites [8]. ILI infection and complications are particularly dangerous in the elderly population, due to the phenomenon of immunosenescence or age-associated decline of immune system activity [9,10].

In order for the body to mount a rapid antiviral immune response, it must detect intracellular viral pathogens. RIG-I-like receptors (RLRs), a family of pattern-recognition receptors (DExD/H box RNA helicases), play a key role in the induction of the innate immune response by sensing viral nucleic acid [11]. RLRs are expressed in the cytoplasm of almost all mammalian cells and include RIG-I (retinoic acid-inducible gene 1, also known as DDX58), MDA5 (melanoma differentiation-associated protein 5, also known as IFIH1), and LGP2 (laboratory of genetics and physiology, DHX58) receptors. Current evidence suggests that RIG-1 and MDA5 can recognize different nucleic acids patterns and capture different byproducts of viral nucleic acid replication and metabolism. RIG-1 has been reported to recognize 5′ phosphorylated short double-stranded RNA (5′ ppp-dsRNA) and the U/A-rich 3′ regions of viral RNA, while MDA5 has been reported to recognize long dsRNA [12,13]. This differential nucleic acid-sensing capacity allows RIG-1 and MAD5 to detect a wide spectrum of infectious viruses. Activation of RIG-1 and MAD5 leads to activation of interferon-I α/β (IFN-I α/β) with their downstream gene network, collectively referred to as interferon-stimulated genes (ISG), such as ISG15 and MX1 [14,15,16]. Both ISG15 and MX1 have been shown to protect against several viruses including influenza infection [17,18,19].

Another essential component of the innate immune response is NK cells, a type of effector cytotoxic lymphocyte. NK cells can engage and lyse cells expressing foreign antigens such as virally-infected and tumor cells [20,21,22,23,24]. The pivotal role of NK cells in controlling various viral infections has been demonstrated for the cytomegalovirus (CMV) [25], hepatitis C virus infection [26], HIV infection [27,28], and viral infection of the lungs [29]. Some of the mechanisms implicated in NK cell-mediated target cell lysis include the release of perforin and granzymes into target cells, the activation of death receptors and induction of apoptosis in target cells, and the release of several pro-inflammatory cytokines [30,31]. It is well established that aging is associated with a decline in NK cell activity concomitant with a reduction of perforin and granzyme expression, which has been proposed as a plausible mechanism for the increased susceptibility of the geriatric population to viral infections and carcinogens [32,33,34].

The natural arabinoxylan rice bran product called Biobran/MGN-3 has been studied as an immunomodulator as well as a beneficial product for aged individuals. Biobran/MGN-3 has been extensively studied for its ability to enhance NK cell activity in both experimental animals and humans [35,36,37,38]. Interestingly, Biobran/MGN-3 has also been shown to counteract age-associated decline of NK cell activity in aged mice [39] and in the geriatric population [40]. Furthermore, Biobran/MGN-3 has been reported to induce potent immunomodulatory effects. For example, in vitro studies of human monocyte-derived dendritic cells, phagocytic cells, and peripheral blood lymphocytes have shown that Biobran/MGN-3 can modulate the production of cytokines such as tumor necrosis factor-α (TNF-α), interferon-gamma (IFN-γ) and -lambda (IFN-λ), and interleukin-2 (IL-2) and IL-12 [36,37,41,42,43]. The potential use of Biobran/MGN-3 against various types of viral infections such as HIV and HCV has also been previously investigated [44,45,46], and Biobran/MGN-3 has been shown to exhibit a psychoneuroimmune modulatory effect by enhancing health-related quality of life in healthy older adults [47] and in cancer patients [48,49].

The well-documented immunomodulatory effect of Biobran/MGN-3 and its capacity to restore the age-associated decline of NK cell activity prompted us to investigate its prophylactic efficacy against virus-induced ILI. To gain insight into the involved mechanisms, we assessed NK cell activity in the studied subjects as well as the expression levels of intracellular RIG-1, MDA5, and the downstream genes ISG15 and MX1 in lung epithelial BEAS-2B cells.

## 2. Subjects and Methods

### 2.1. Trial Design

**Figure 1:**
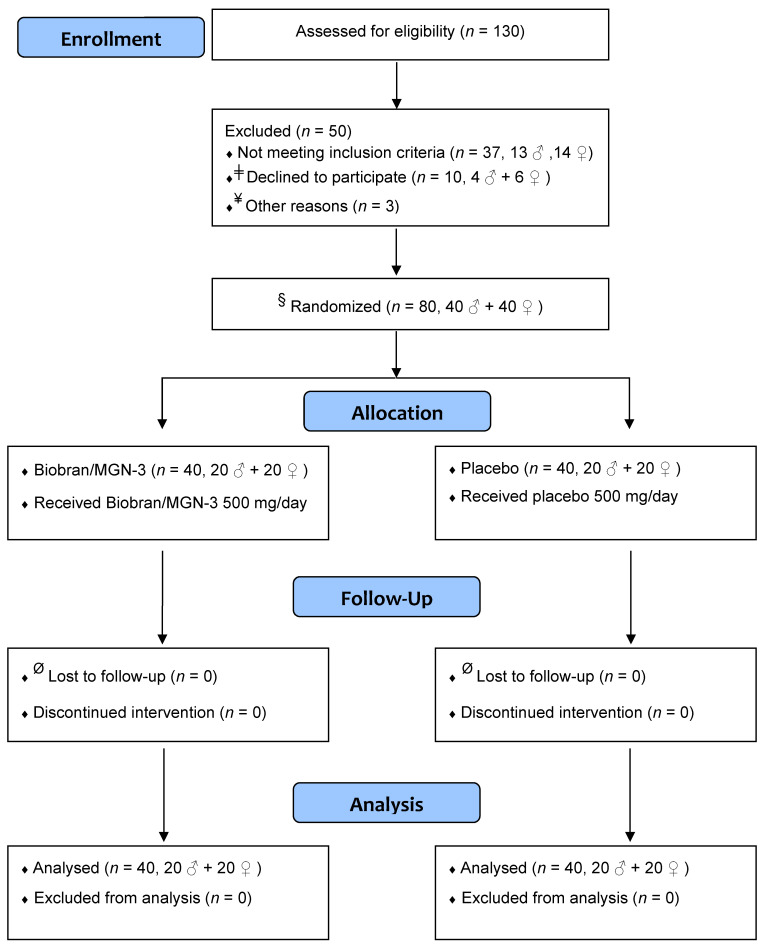
Simple flow diagram showing different stages of the clinical trial. ^ǂ^ Declined to participate when informed that the intervention included unlabeled coded sachets. ^¥^ Not enrolled to adjust males/females ratio to 1. ^§^ The principal investigator was not involved in the randomization or allocation procedure, which was performed by a statistician ignorant of the study. ^Ø^ Close monitoring, daily follow-up of subjects, and the relatively short period of the study (3 months) resulted in the absence of any dropouts.

A double-blind placebo-controlled design was utilized in the current clinical trial to assess the effect of Biobran/MGN-3 on the incidence of ILI in older adults. Figure 1 depicts a simple flow diagram of the clinical trial. The study spanned the time period from November 2018 until the end of February 2019, a period with known peak incidence of ILI attacks in Egypt [8]. The study protocol conformed to the ethical guidelines of the 1975 Declaration of Helsinki and was approved by the Institutional Review Board, Zagazig University Hospital, Faculty of Medicine (IRB# 1507/1/6/2017). The clinical trial was registered at clinicaltrials.gov with the ID# NCT04646980 on 30 November 2020.

### 2.2. Participants

A total of 130 subjects ≥ 56 years was initially recruited from the visitors of outpatient clinics at Zagazig University Hospitals, Egypt, and assessed for eligibility to participate in the clinical trial. Only local residents of Zagazig city were recruited in order to facilitate follow-up home visits. Thirty-seven subjects (*n* = 37, 19 ♀, 18 ♂) did not meet the inclusion criteria and 10 subjects refused to participate when they were informed that the intervention included unlabeled sachets. Three randomly chosen males by a statistician ignorant to the study were not enrolled in the study to adjust the males/females ratio to 1. Finally, eighty apparently healthy older adults ≥ 56 years, 40 males and 40 females, were enrolled in the study. Subjects ≥ 56 years were recruited to the study in order to meet the WHO definition of old age in African nations [50]. In addition, this age is close to public service retirement in Egyptian society, which is associated with significant social, mental, and psychological stress and therefore could be associated with significant decline in NK cell activity. Close monitoring, daily follow-up of subjects, and the relatively short period of the study (3 months) resulted in the absence of any dropouts. 

### 2.3. Inclusion and Exclusion Criteria

Inclusion criteria included older adults ≥ 56 years who were local residents of Zagazig, Egypt, at the time of the study and who were willing to participate voluntarily in the study. Local residents were chosen to ensure close monitoring and to reduce dropout rate. Exclusion criteria included subjects who had taken an influenza vaccine, cortisone, or any other immunosuppressive agents such as radiation or chemotherapy at the time of the study. Exclusion criteria also included subjects with a history of chronic infections (e.g., TB), malignancies, auto-immune disorders, liver or kidney failure, and major psychological insults for probable un-cooperation with instruction. 

### 2.4. Consent

Informed consents were obtained from all participants after explaining the study purpose and design. 

### 2.5. Randomization and Allocation Concealment

Males (*n* = 40) and females (*n* = 40) were randomly allocated into the two study arms at a ratio of 1:1 to yield 40 subjects/arm (20 males and 20 females). Randomization and allocation were performed by a statistician ignorant of the study using SPSS software.

### 2.6. Intervention

Biobran/MGN-3 or placebo, 500 mg/day for 3 months, was used in the form of powder enclosed in sachets. Biobran/MGN-3 is a denatured hemicellulose whose main chemical structure is an arabinoxylan with a xylose in its main chain and an arabinose polymer in its side chain. It is obtained by reacting rice bran hemicellulose with several carbohydrate-hydrolyzing enzymes from Shiitake mushrooms, yielding a polysaccharide that contains β-1,3-glucans and activated hemicelluloses [44].

### 2.7. Ingredients of Biobran/MGN-3 Sachets

The Biobran/MGN-3 sachet’s ingredients included Biobran: 500 mg, hydroxypropyl distarch phosphate: 280 mg, dextrin: 200 mg, tricalcium phosphate: 20 mg, and maltitol: 1000 mg. The placebo sachet’s ingredients included maltitol: 1000 mg, hydroxypropyl distarch phosphate: 780 mg, dextrin: 200 mg, and tricalcium phosphate: 20 mg.

### 2.8. Blinding

To ensure blinding, sachets of Biobran/MGN-3 and placebo, kindly provided by Daiwa Pharmaceuticals Co., Ltd., Tokyo, Japan, were indistinguishable except by imprinted letter code. The manufactured company provided the Biobran/MGN-3 and placebo powder in a formulation that could not be differentiated by the shape, smell, color, or even taste. Both participants and health care provider were blinded to the intervention during all phases of the study. The code was only revealed after complete analysis of the results.

### 2.9. Follow-Up

Participants were instructed to call upon the health care giver upon the incidence of any complaint, ailment, fever, cough, or diarrhea. In addition to daily follow-up by phone, participants were offered weekly home visits to closely monitor their health status. No vitamins or any over-the-counter drugs were allowed during the study without consultation. ILI diagnosis was documented by the incidence of acute respiratory illness, cough with temperature ≥ 38 °C [3]. Diagnosed ILI cases received the proper treatment by the health care giver.

### 2.10. Outcome

The primary outcomes of the current trial included (i) the incidence of ILI infection in the Biobran/MGN-3 arm compared to the placebo arm and (ii) confirmation of the effect of Biobran/MGN-3 on NK cell activity. ILI diagnosis was made by documenting the incidence of acute respiratory illness, cough and temperature of ≥38 °C [3]. The incidence rate was calculated by dividing the number of incident ILI cases by the number of participants in each group. The incidence density was calculated by dividing the number of incident cases by the total person-time at risk [51].

The secondary outcome included the incidence of any clinically reported or laboratory-observed adverse effect. 

### 2.11. Sample Size

To our knowledge, the prophylactic effect of Biobran/MGN-3 on reducing the incidence of ILI was not previously studied. As such, the current pilot clinical trial was conducted with 80 subjects to investigate the efficacy of Biobran/MGN-3 in reducing ILI incidence. The effect of Biobran/MGN-3 on NK cell activity was measured in six randomly selected subjects from each group because this number was shown to be sufficient for detecting a significant difference between Biobran/MGN-3 and the placebo [40].

### 2.12. Laboratory Investigations

Blood samples (5 cc) were collected from a randomly selected six subjects in each group for laboratory investigation. Alanine aminotransferase (ALT/SGPT) and aspartate aminotransferase (AST/SGOT) were used to monitor liver functions, and serum uric acid was used to monitor kidney function. The following hematological parameters, red blood cell count (RBC), total hemoglobin (Hb), mean corpuscular hemoglobin (MCH), hematocrit (HCT), mean corpuscular volume (MCV), and total and differential white blood cells count, were also assessed.

### 2.13. Degranulation Assay for NK Cell Activity

The activity of NK cells was measured using the degranulation assay as previously described by Elsaid et al. [40]. Heparinated blood (100 μL) was collected to examine NK activity under 4 conditions: (1) unstimulated condition (RPMI 1640 medium alone); (2) stimulated condition (combination of phorbol-12-myristate-13-acetate (PMA, 50 ng/mL, Sigma, St. Louis, MO, USA) and Ca^2+^ ionophore (ionomycin, 250 ng/mL, Sigma, St. Louis, MO, USA); (3) IgG isotypic staining negative control; and (4) positive control (PMA/ionomycin plus cytochalasin (5 µg/mL, Sigma, St. Louis, MO, USA). For staining, samples were incubated with FITC-labeled mouse anti-human CD-107a (clone H4A3, BD Bioscience, San Jose, CA, USA) for 5 h at 37 °C under 5% (*v*/*v*) CO_2_. One hour later, monensin (GolgiStop, BD Bioscience, San Jose, CA, USA) was added to a final concentration of 6 µg/mL. To stain the IgG isotypic negative control, samples were incubated with mouse anti-human FITC-labeled IgG AB (clone G18-145, BD Bioscience, San Jose, CA, USA) [52]. At the end of incubation, RBCs were lysed using BD PharmLyse (BD Bioscience). Cells were spun down by centrifugation (300× *g*, 5 min), washed once, and re-suspended with 5 µL of PE-labeled mouse anti-human CD56 AB clone R19-760 (NCAM-1, BD Bioscience) and 5 µL of PerCP-labeled mouse anti-human CD3 clone SK7 (BD Bioscience) in a final volume of 100 µL staining buffer for 15 min at 37 °C. Cells were collected with centrifugation (300× *g*, 5 min), washed once, and re-suspended in 100 μL FACS buffer. BD FACS Calibur with CellQuest software was used for analysis (BD Bioscience, San Jose, CA, USA).

### 2.14. Cell Culture and Flow Cytometry

BEAS-2B cells (ATCC) were grown in bronchial epithelial growth medium using the BEGM Bullet kit (Lonza, Walkersville, MD, USA) on collagen-coated (5 g/cm^2^) plates and maintained at 37 °C, 5% CO_2_. Cells were then seeded in 6-well plates and grown until 90% confluent, then incubated with either Biobran/MGN-3, 100 μg/mL (Daiwa Technologies, Japan), for 72 h or PBS (control). After harvesting, cells were washed with PBS, permeablized and fixed using BD cytofix/cytoperm buffer as recommended by the manufacturer. Cells were then spun down, washed, and re-suspended in 100 µL of the first block solution with 10% rabbit serum for 30 min at room temperature. Staining was performed by incubating the cells for 15 min at 37 °C with 5 µL of either fluorescent-labeled monoclonal anti-RIG-1 PE (catalog # sc-376845 PE, Santa Cruz Biotechnology, Dallas, TX, USA), monoclonal rabbit anti-human MX1 AB (catalogue # orb228747, Biorybt, San Francisco, CA, USA), monoclonal rabbit anti-human MDA5 AB, clone # 33H12L34 (catalog # 700360, ThermoFisher Scientific, Rockford, IL, USA), monoclonal rabbit anti-human anti-ISG15 AB (catalog # ab133346, Abcam, Cambridge, MA, USA), or the IgG isotype-matched control AB (negative control). For RIG-1, cells were washed once in FACS buffer, collected by centrifugation, re-suspended in FACS buffer, and analyzed. For 2 ry AB staining of MDA5, MX1, and ISG15, cells were washed in permeablized/fixed BD cytofix/cytoperm buffer, collected by centrifugation, then re-suspended in staining buffer with 10% goat serum (secondary block) for 30 min at room temperature. Finally, cells were incubated with PE-conjugated goat anti-rabbit IgG secondary antibody (catalog # F0110, R&D Systems, Minneapolis, MN, USA) at 10 µL/10^6^ cells. Data acquisition and analysis were performed using FACS Calibur (BD Biosciences) and BD Cell Quest software (BD Biosciences). All cell culture and flow experiments were performed in triplicate.

### 2.15. Statistical Analysis

Continuous variables were presented as mean ± standard deviation (SD). Data normality was checked using the Shapiro–Wilk test. Comparison of quantitative variables between groups was performed using a two-tailed Student’s *t*-test, whereas comparison within groups (post-intervention vs. basal levels) was performed using paired *t*-test. Comparison of the incidence rates and density between Biobran/MGN-3 and placebo groups was performed using the Fisher’s exact test. Level of significance of all statistical tests was set at an alpha level of significance ≤ 0.05. OpenEpi, version 3.01, and SPSS, version 20, were used for statistical analyses [53,54].

## 3. Results

### 3.1. Biobran/MGN-3 Did Not Adversely Affect the Hematological, Liver, and Kidney Functions

The average age of participants in the Biobran/MGN-3 group (61.5 ± 5.6 years) was nearly equal to that in the placebo group (62.6 years ± 5.7). Results depicted in Table 1 show that all basal level parameters of hematological, liver, and kidney functions were not significantly different between the two groups. This indicates that Biobran/MGN-3 did not adversely affect any of the hematological, liver, or renal functions. This finding is consistent with previous studies demonstrating that Biobran/MGN-3 is safe without any adverse effects [46,49,55,56].

### 3.2. Biobran/MGN-3 Supplementation Significantly Reduced the ILI Incidence Rate and Density

**Figure 2:**
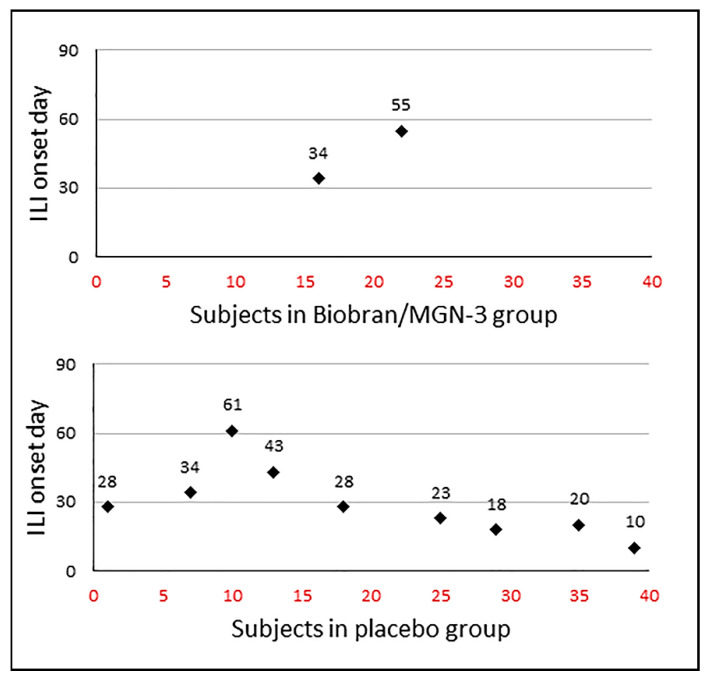
ILI incidence density distribution among the MGN-3/Biobran and placebo groups. The x-axis represents the patient number and y-axis represents the day of onset of ILI.

Figure 2 shows the incident cases and the day of ILI onset in the two groups. The incidence rate in the Biobran/MGN-3 group was 5% (2/40), which was significantly lower than the incidence rate in the placebo group which reached 22.5% (9/40) (*p* = 0.048 using a two-tailed Fisher’s exact test). The risk of ILI infection in the Biobran/MGN-3 group was estimated to be 18.2% (CI = 3.9 − 48.9), which was significantly lower than the 55.1% (CI = 43.4 − 66.3) value for the placebo group (*p* < 0.05). The risk ratio of ILI infection in the Biobran/MGN-3 group compared to the placebo group was estimated to be 0.33, using the Taylor series approximation. The estimated preventive fraction in the Biobran/MGN-3 group was almost 82% (95% CI = 9.9% − 96.4%). Similarly, significant results were obtained when comparing the incidence density rates in the two groups. The incidence density rate in the Biobran/MGN-3 group was estimated to be 0.57 cases per 1000 person-days (95% CI = 0.06 − 2.06) compared to 2.95 cases per 1000 person-days (95% CI = 1.34 − 5.59) in the placebo group (*p* = 0.038 using a two-tailed Fisher’s exact test). The risk ratio of ILI infection in the Biobran/MGN-3 group compared to the placebo group was estimated to be 0.19 using the Byar method. The preventive fraction in the Biobran/MGN-3 group was estimated to be 81% (95% CI = 10.5% − 95.8%).

### 3.3. Biobran/MGN-3 Specifically Enhanced NK Cell Activity but Not NKT Activity

Supplementation with Biobran/MGN-3 at 500 mg/day significantly enhanced NK cell activity, as suggested by the percentage of CD107a-expressing NK cells upon stimulation with PMA/ionomycin compared to basal levels as well as to the placebo group (Table 2). This effect of Biobran/MGN-3 was specific to NK cells; we did not observe a similar effect in NKT cells.

### 3.4. Biobran/MGN-3 Upregulates the Expression of RIG-1, MDA5, ISG15, and MX1 in Pulmonary Epithelial BEAS-2B Cells

**Figure 3:**
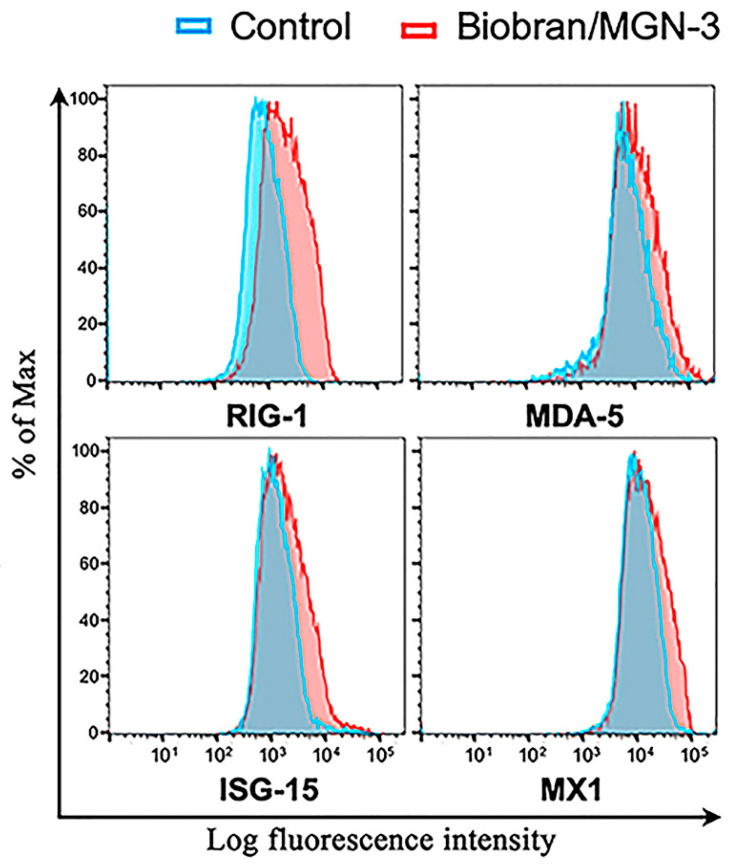
Upregulation of intracellular antiviral sensors RIG-1, MDA5, and their downstream responsive genes ISG15 and MX1 in the human lung BEAS-2B epithelial cells when exposed to Biobran/MGN-3 (100 μg/mL) for 72 h. Histograms depict gene expression as determined by the log fluorescent intensity on the x-axis, whereas % of cells normalized to the maximal number on the y-axis. At higher than average fluorescent intensity, higher % of cells exist due to shifting of the curve to the right. Each histogram is representative of three flow cytometry experiments.

Figure 3 shows that Biobran/MGN-3 exposure at a concentration of 100 μg/mL, which is comparable to its plasma concentration after daily intake of 500 mg, significantly upregulated the RIG-1 and MDA5 expression in BEAS-2B pulmonary epithelial cells compared to its expression in the control conditions. Biobran/MGN-3 exposure also enhanced the expression of MX1 and ISG15, both belonging to ISG downstream of RIG-1 and MDA5.

## 4. Discussion

The current study demonstrates the safety and prophylactic efficacy of Biobran/MGN-3 in reducing ILI incidence rate and incidence density. Supplementation of Biobran/MGN-3 (500 mg/day) for 3 months in our study did not result in any subjectively reported or laboratory-indicated side effects. This result was consistent with safety reports obtained from several clinical studies, with some of them reporting the safe use of Biobran/MGN-3 at doses of 1000, 2000, and 3000 mg/kg/day [46,49,55,56]. In addition to being safe, supplementation with Biobran/MGN-3 significantly reduced the incidence rate and risk of ILI infection from 25.5% and 55% in the placebo group to 5% and 18%, respectively. In support of our results, it was recently reported that ingestion of arabinoxylan (10 g/day for 5 weeks), the main constituent of Biobran/MGN-3, has reduced the incidence of common cold and boosted the vaccine-mediated seroprotection against influenza A H1N1 compared to the control group in seniors aged 50–79 years [57]. In our study, the preventable fraction attributed to Biobran/MGN-3 supplementation was approximately 80%, as calculated from a reduction of either incidence rate (considering persons only) or incidence density rate (considering person-time).

Our finding that Biobran/MGN-3 supplementation significantly reduced ILI incidence suggests that it could be protective against influenza as well as other viruses known to be associated with ILI. The 80%-prevented fraction observed in the Biobran/MGN-3 group, given that influenza viruses were isolated from only 13% of ILI cases in Egypt [8], supports the conclusion that Biobran/MGN-3 has broad antiviral activity. Earlier studies showed that Biobran/MGN-3 supplementation was associated with reduced risk of infection and duration of the common cold among geriatric subjects aged 70–95 years [45]. Interestingly, the antiviral activity of Biobran/MGN-3 has been demonstrated for non-respiratory viruses as well. For example, Biobran/MGN-3 has been shown to inhibit replication of the HIV virus [44] and to reduce the viral load in chronic hepatitis C patients [46].

The prophylactic activity against a broad spectrum of viruses suggests that Biobran/MGN-3 could directly enhance the innate immune system. Therefore, we investigated the effect of Biobran/MGN-3 on two components of the innate immune system, namely NK cells and the intracellular viral nucleic acid receptors RIG-1 and MDA5. Our results demonstrated that Biobran/MGN-3 supplementation significantly upregulated NK cell activity. These results were consistent with other studies involving tissue cultures, experimental animals, and humans [35,36,37,38,39,40]. NK cells play an essential role in the innate immune response through their capability to eliminate virally-infected cells, without the need for prior sensitization to the viral antigen [20,21,58]. Impaired NK function has been shown to be associated with increased risk of viral infections including the influenza virus [59], hepatitis C virus [60], and herpes viruses [61].

The effect of Biobran/MGN-3 on the expression levels of RIG-1 and MDA5, the key intracellular sensor of viral nucleic acids, was also studied using lung epithelial cells. Biobran/MGN-3 ingestion upregulated the expression of both receptors, which was functionally relevant, as confirmed by the upregulated expression of their downstream targets ISG15 and MX1. RIG-I was found to be activated by positive- and negative-stranded RNA viruses such as influenza, Rift Valley fever, measles, Ebola, vesicular stomatitis, and hepatitis C viruses [62], whereas MDA5 was found to be activated by picornavirus, arteriviruses, hepatitis D, encephalomyocarditis, and Kaposi’s sarcoma-associated herpesvirus [63,64,65,66]. Our finding that ISG15 has been upregulated is important because it has been shown to inhibit replication of several viruses such as the influenza virus, vaccinia virus, human immunodeficiency virus 1, vesicular stomatitis virus, and dengue virus [18,67]. Homozygous ISG15-knockout mice exhibited high mortality rate when infected with influenza B or A viruses [18,19]. Similarly, MX1 upregulation is also important because it has been linked to the high resistance of the A2G mouse inbred strain to influenza infection [17]. Collectively, Biobran/MGN-3 was found to upregulate the expression of RIG-1 and MDA5 intracellular receptors and their downstream ISG15 and MX1, with known direct and indirect broad antiviral activity.

The finding of the current study has several implications. One implication is related to NK induction. Several lines of research have focused on NK cells as a prophylactic and/or therapeutic agent against virus infection and cancers [20,21,22,23,24]. In this regard, Bioran/MGN-3 could be a novel agent for modulating NK activity. Another implication is related to the induction of RIG-1 and MDA-5. A crucial role for MDA5 and RIG-1 in sensing and mounting effective antiviral response against SARS-Cov-2 has recently been reported [68,69]. Therefore, it is plausible to propose that Biobran/MGN-3 could counteract COVID-19 infection. It is worth mentioning that Biobran/MGN-3 is a notable biological response modifier in that it does not exhibit hyporesponsiveness [48], making it a unique and attractive agent for long-term preventive and/or therapeutic purposes.

The strengths of our study stem from (1) its elimination of biased assessment because of its design as a prospective double-blind placebo-controlled clinical trial, (2) its emphasis on the prophylactic efficacy and safety of Biobran/MGN-3, and (3) its inclination towards older individuals (average age of 60 years) who are more susceptible to infection and cancers. The study is nevertheless limited in its relatively small sample size, assessment of RIG-1, MDA5, ISG-15, and MX1, which was performed in tissue culture, and lack of disease severity assessment after infection. The results are very encouraging and warrant replications with larger sample sizes and more detailed assessments.

## 5. Conclusions and Future Direction

Our data suggest that Biobran/MGN-3 is a safe, non-toxic, and effective antiviral agent whose activity stems from its potent stimulating effect on several components of the innate immune system. Biobran/MGN-3 supplementation was found to upregulate NK cell activity and the expression levels of RIG-1 and MDA5, as well as their downstream ISG15 and MX1 genes, known for their broad antiviral effects. Biobran/MGN-3 could be a novel antiviral agent that is well suited for long-term preventive purposes. Our future work includes inclusion of a larger sample size and expands our objective to include antiviral effects against COVID-19.

## Figures and Tables

**Table 1:** Comparison of hematological indices and liver and kidney functions within groups (before and after treatment) and between groups (Biobran/MGN-3 and placebo groups).

Treatment	Parameter	Baseline	Post-Treatment	*p*-Value §
Placebo ǂ	RBCs (×10^6^/µL)	4.3 (0.5)	4.1 (0.5)	0.58
	HB (g/dL)	11.3 (1.5)	13.8 (1.4)	0.40
	HCT %	35.7 (4.0)	36.7 (3.5)	0.51
	MCHC (g/dL)	27.3 (2.7)	37.6 (0.7)	0.80
	WBC (×10^3^/µL)	5.7 (0.9)	6.1 (1.2)	0.76
	Neutrophils %	56.1 (14.5)	57.3 (11.6)	0.32
	Lymphocytes %	33.3 (10.2)	39.5 (9.8)	0.23
	Platelets (×10^3^/µL)	218.8 (48.4)	231.5 (48.0)	0.19
	ALP (U/L)	185.5 (51.3)	183.2 (50.1)	0.66
	ALT (U/L)	22.0 (9.3)	20.3 (8.4)	0.15
	AST (U/L)	17.7 (4.6)	18.3 (5.8)	0.07
	UA (mg/dL)	7.4 (2.9)	8.2 (3.8)	0.51
Biobran/MGN-3 ǂ (500 mg/day) for 3 months	RBCs (×10^6^/µL)	4.8 (0.5)	4.7 (0.6)	0.18
	HB (g/dL)	12.1 (1.7)	13.9 (1.6)	0.93
	HCT %	37.4 (4.4)	36.9 (3.1)	0.6
	MCHC (g/dL)	32.3 (0.9)	37.4 (1.5)	0.46
	WBC (×10^3^/µL)	6.8 (1.8)	6.7 (1.4)	0.68
	Neutrophils %	57.3 (14.3)	60.7 (7.3)	0.37
	Lymphocytes %	32.5 (7.9)	38.8 (8.5)	0.24
	Platelets (×10^3^/µL)	187.3 (37.1)	208.8 (16.9)	0.12
	ALP (U/L)	213.0 (43.0)	195.2 (35.1)	0.58
	ALT (U/L)	22.0 (7.2)	19.3 (6.6)	0.38
	AST (U/L)	23.8 (11.0)	20.2 (8.8)	0.26
	UA (mg/dL)	5.9 (2.5)	6.7 (1.8)	0.33

§ *p*-values represent comparison between post-treatment and baseline values and were determined using paired *t*-test because all data were normally distributed. All comparisons yielded non-significant differences between the post-treatment and the basal levels. ǂ Comparison between placebo and Biobran/MGN-3 groups yielded non-significant differences with regards to all variables. *p* ≤ 0.05 was considered statistically significant. RBC, red blood cell; Hb, hemoglobin; HCT, hematocrit; MCV, mean corpuscular volume; MCH, mean corpuscular hemoglobin; WBC, white blood cell; ALT, alanine aminotransferase; AST, aspartate aminotransferase; UA, uric acid.

**Table 2:** Comparison of NK, NKT, and CD-107a-expressing NK and NKT cells within groups (before and after treatment) and between groups (Biobran/MGN-3 and placebo groups).

Treatment	Parameter	Baseline	Post-Treatment	*p*-Value §
Placebo	NK	5.3 (1.9) ǂ	6.0 (1.7) ǂ	0.62
	NKT	4.5 (1.6) ǂ	5.6 (2.4) ǂ	0.44
	NK CD-107 a	45.3 (12.0) ǂ	50.8 (19.5) ¥	0.38
	NKT CD-107 a	67.9 (15.6) ǂ	75.5 (22.3) ǂ	0.23
Biobran/MGN-3 (500 mg/day) for 3 months	NK	6.1 (2.6) ǂ	6.7 (1.7) ǂ	0.60
	NKT	3.1 (0.6) ǂ	4.6 (2.5) ǂ	0.25
	NK CD-107 a	49.5 (10.4) ǂ	75.2 (6.6) ¥	0.004 *
	NKT CD-107 a	70.6 (10.1) ǂ	76.9 (9.8) ǂ	0.25

§ *p*-values represent comparison between post-treatment and baseline values and were determined using paired *t*-test because all data were normally distributed. *p* ≤ 0.05 was considered statistically significant. * The proportion of PMA/ionomycin-stimulated NK cells expressing CD-107 a was significantly higher at post-treatment compared to the basal levels. ¥ Values were significantly higher in the Biobran/MGN-3 group compared to the placebo group (*p* = 0.026). ǂ Values were not statistically different between the placebo and the Biobran/MGN-3 groups. NK, natural killer cells; NKT, natural killer T-cells; NK CD-107 a, PMA/ionomycin-stimulated NK cells expressing CD-107 a; NKT CD-107 a, PMA/ionomycin-stimulated NKT cells expressing CD-107 a.

## Data Availability

Data are available upon request from the corresponding author or Ghoneum M.
